# Toluene adsorption on porous Cu–BDC@OAC composite at various operating conditions: optimization by response surface methodology

**DOI:** 10.1039/d0ra06578a

**Published:** 2020-09-29

**Authors:** Amir Hossein Khoshakhlagh, Farideh Golbabaei, Mojtaba Beygzadeh, Francisco Carrasco-Marín, Seyed Jamaleddin Shahtaheri

**Affiliations:** Department of Occupational Health Engineering, School of Public Health, Tehran University of Medical Sciences Tehran Iran shahtaheri@tums.ac.ir +98-2188951390; Department of Energy, Materials & Energy Research Center P. O. Box: 14155-4777 Tehran Iran m.beygzadeh@merc.ac.ir +98-26-36280040-9; Carbon Materials Research Group, Faculty of Science, University of Granada Avda. Fuente Nueva s/n Spain

## Abstract

The work presented here describes the synthesis of Cu–BDC MOF (BDC = 1,4-benzenedicarboxylate) based on oxidized activated carbon (microporous Cu–BDC@OAC composite) using an *in situ* method. The adsorbents (oxidized activated carbon (OAC), Cu–BDC and microporous Cu–BDC@OAC composite) were characterized by XRD, FTIR, SEM, EDS and BET techniques. Optimization of operating parameters affecting the efficiency of adsorption capacity, including adsorbent mass, flow rate, concentration, relative humidity and temperature, was carried out by central composite design (CCD) of the response surface methodology (RSM). An adsorbent mass of 60 mg, a flow rate of 90 mL min^−1^, the concentration of toluene (500 ppm), the relative humidity of 30% and a temperature of 26 °C were found to be the optimized process conditions. The maximum adsorption capacity for toluene onto Cu–BDC@OAC composite was 222.811 mg g^−1^, which increased by almost 12% and 50% compared with pure Cu–BDC and oxidized AC, respectively. The presence of micropores enhances the dynamic adsorption capacity of toluene. The regeneration of the composite was still up to 78% after three consecutive adsorption–desorption cycles. According to the obtained adsorbent parameters, microporous Cu–BDC@OAC was shown to be a promising adsorbent for the removal of volatile organic compounds.

## Abstract 2

The work presented here describes the synthesis of Cu–BDC MOF (BDC = 1,4-benzenedicarboxylate) based on oxidized activated carbon (microporous Cu–BDC@OAC composite) using an *in situ* method.

## Graphical abstract

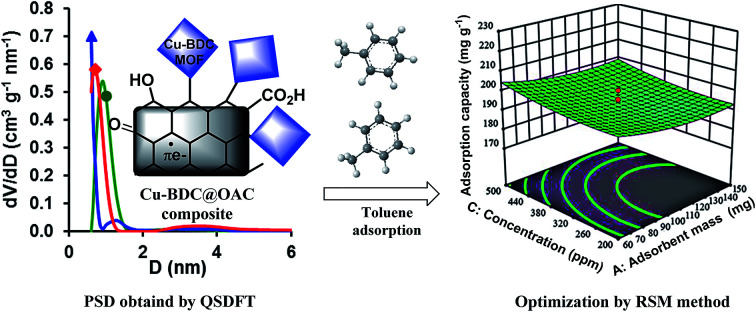

## 1. Introduction

Volatile organic compounds (VOCs) are organic chemicals that have a high vapor pressure at room temperature and a low boiling point in the range of 50–100 °C to 240–260 °C. The sources of VOCs are diverse and include burning fuel, such as gasoline, wood, coal, or natural gas, or a variety of consumer products such as cigarettes, solvents, pesticides, *etc.* Despite being valuable for many industrial applications, volatile organic compounds (VOCs) are also a health hazard to humans.^1^

Toluene is one of the most common VOCs, that is widely used in the production of fuels and other industrial products.^2,3^ Various studies have reported that exposure to toluene has major effects on human health, causing diseases such as encephalopathy, headaches and loss of coordination.^4–6^ The OSHA's (occupational safety and health administration) permissible exposure limit (PEL) standard for toluene as an 8 hour time-weighted average (TWA) limit is 200 ppm, with a maximum limit of 300 ppm (not to be exceeded for more than 10 minutes in any 8 hour TWA); in quantities above 500 ppm it becomes immediately dangerous to life or health (IDLH).^7^

Hence, there is a pressing need to remove VOCs from the atmosphere. Various physical, chemical and biological removal techniques have been widely used such as adsorption,^8,9^ oxidation,^1,9^ bio-filtration,^10,11^ biodegradation,^12^ bio-reactor removal^13,14^ and catalytic combustion.^15^ Among the above technologies, adsorption is a good candidate method for air purification, as it is efficient, low cost and a simple technique. Various porous materials have been studied as adsorbents for the abatement of VOCs from contaminated air. They consist of activated carbon, carbon nanotubes, zeolites, graphene and metal–organic frameworks (MOFs).^8,9,16,17^

Metal–organic frameworks (MOFs) are known to have explicit pore structures, a high specific surface area and a high pore volume, which makes them good candidates for many applications, such as gas storage and adsorption.^18,19^ Compared to other traditional porous materials like activated carbon, MOFs have excellent advantages for the removal of VOCs due to high specific surface area, diverse structural composition, a large number of active adsorption sites and cation–π bonding through electrostatic attraction with a benzene ring.^20^ A large number of MOF materials are synthesized for VOCs adsorption, which were design to display high selectivity or capacity.^20^ However, MOFs have a low resistance to high humidity and high temperature, which significantly limits their application in a humid environment.^21^ To alleviate this, the water stability of MOFs was enhanced by optimized synthetic methods which produced MOFs composites, including MOF–silica, MOF@zeolite, MOF@organic polymers, MOF@CNT and MOF@carbon.^21^

The most widely used adsorbent for the adsorption process in industrial wastewater treatment systems is activated carbon due to its large specific surface area and high pore volumes.^22,23^ On the other hand, the modification of activated carbon and the preparation of new composite adsorbent materials from modified activated carbon are necessary.^24^ Therefore, researches have been continued for the preparation of new alternative adsorbents having reasonable adsorption efficiencies.^25^

Also, activated carbon has been commonly used as adsorbents for volatile organic compounds (VOCs), due to the hydrophobic surface that is appropriate for adsorption of organic compounds.^26^ In recent years, it has attracted attention as a low cost solution with a number of useful features (chemisorption, high surface area, material hardness, *etc.*).^27,28^ Its surface area range is between 800 and 1200 m^2^ g^−1^. Generally, activated carbon can be used in a fixed, moving, or fluidized bed system. The advantages of fixed beds include low cost and longer packing life.^29^ One of the essential factors for the removal of compounds is surface chemistry. The lack of functional groups on the external surface of the activated carbon (AC) can limit its practical use. Hence, a modification of its surface chemistry was a key point for the work presented here. Oxidized activated carbon (OAC), produced by the introduction of independent monoprotic functional groups on AC surfaces, such as carboxyls (–COOH) can be a favorable solution. Carboxyl groups are affiliated as common oxygen functions on activated carbons. The presence of surface oxygens can influence the adsorption properties of AC, as a result of the formation of additional and diverse binding sites.^3,26^ Chemical or thermal treatments can be used to manipulate the proportion of oxygen surface groups. In contrast to heating, oxidation/activation can be used for increasing the number of oxygen surface groups.^26^

Cu–BDC MOF contains copper nodes interconnected by 1,4-benzene dicarboxylate (BDC) coordinated as 2D layers within the bulk crystal. The Cu–BDC MOF is often water stable and can be used as an adsorbent to remove pollutants and gas storage.^19,30^ By combining with other materials, the characteristics of MOFs can be enhanced to a greater extent for improved functionality/stability, simplicity of preparation and selectivity of operation. For example, the composite Cu-BTC@GO (comprising of Cu-BTC and graphite oxide) improves toluene adsorption capacity.^31^ As it was mentioned, the MOF or modified MOF material proves magnificent adsorptive toluene performance in the adsorption system.

Furthermore, some of the MOFs have been synthesized and studied under the specific attention of avoiding exposure to air, so enhancing the stability and suitability of MOF materials is needed more research field. The mixture of a MOF and AC can improve performance because most carbon-based materials inherently possess excellent stabilities towards water/vapor. In a study conducted for adsorptive desulfurization, MOF-5@AC was synthesized. The results showed that the composite was an excellent material for adsorptive desulfurization.^20^

Further experimental research can provide insights on the effects of applying suitable conditions of critical operating parameters, including concentration, flow rate, temperature and relative humidity.^3^ The optimal experimentation design will have to possess the following three features: (1) cost reduction; (2) accommodation of several parameters, for example, process, variety and separate parameters; (3) optimization of designs can be done when the design-space is constrained.^2,32^

There are several types of design for experiments, such as one-factor-at-a-time and the response surface methodology (RSM) design. Taking into account the disadvantages of conventional one-factor-at-a-time optimization method such as requiring more runs for the same precision, time-consuming, high overall costs, failure to estimate interactions and missing optimal settings of parameters, using statistical methods such as response surface methodology (RSM) can be performed to enhance the production of a particular substance by optimization of operational variables.^2,33^ Lately, there has been an enormous increase in the use of RSM in studies.^2,33,34^ Amari *et al.* used central composite design (CCD) as an experimental design for the investigation of toluene adsorption onto acid-activated clay. They studied the effect of temperature, contact time, the mass ratio of liquid/solid and strength of the acid. The optimized proportions of the following variables, *i.e.*, temperature, contact time, the mass ratio of liquid/solid and strength of acid were found to be 96.2 °C, 6.93 h, 5.98 and 32.94%, respectively.^2^ To date, a number of studies have addressed the reduction of toluene just under certain circumstances such as concentration.^3,8,26,35,36^ These studies did not perform an optimization of essential variables regarding the adsorption capacity. The present study thus addresses this knowledge gap with the aim to provide a comprehensive study of the adsorption behavior of toluene under various operational parameters.

As it was mentioned above, among many MOF materials, Cu–BDC MOF is inexpensive and environmentally friendly and also has a uniform pore size and open-pore structure. After Cu–BDC is combined with oxidized activated carbon, the hydrophobicity and specific surface area are increased by synergistic action.^19,30,37^ Therefore, this article aimed to synthesize microporous Cu–BDC@OAC composite from incorporating Cu–BDC MOF into oxidized activated carbon with high specific surface area and porosity to enhance the performance of toluene adsorption at various operating conditions. Also, the other purpose of this study is to present a systematic method to determine what range of essential variables has a higher effect on the VOC adsorption. For achieving this aim, optimization will be done by the response surface methodology. Finally, the reusability of the synthesized composite will be investigated.

## 2. Experimental

### 2.1 Materials

All chemicals used, including toluene, copper(ii) nitrate trihydrate, terephthalic acid, *N*,*N*-dimethylformamide (DMF), nitric acid (65%) and hydrochloric acid (1 M) (Titrisol® concentrated solution) were purchased from Merck Co. (Germany). Commercial activated carbon (815 m^2^ g^−1^) was supplied by Caware Int'l Corp. (Taiwan).

### 2.2 Pretreatment of adsorbents

Samples (activated carbon) were ground using a mortar grinder (RM 200, Retsch Company) and sieved to a mesh of 35–60 (250–500 μm). After that, the ground sample was mixed with distilled water using stirrer for 1 h with a velocity of 150 rpm to remove impurities, the suspension was passed through a standard sieve and washed 3 times using distilled water. Then, the sample was dried in the oven at 105 °C for 24 h to remove the moisture till constant weight. The dried sample was heated at 200 °C for 1 h in a furnace (Carbolite, UK) in the presence of air.^38^ Finally, the samples were maintained in a desiccator for further use.

### 2.3 Synthesis of Cu–BDC MOF

The Cu–BDC MOF was prepared by a previously reported method adapted from the published procedure,^39^ at first, 1 : 1 molar ratio of Cu(NO_3_)_2_·3H_2_O (1.45 g, 6 mmol) and terephthalic acid (1 g, 6 mmol) were mixed in 75 mL DMF. The mixture was stirred for 10 min and after that transferred to a glass reagent bottle heated at 110 °C for 36 h. The bottle was then cooled to room temperature outside the oven. The resulting blue precipitate was filtrated and washed with fresh DMF several times over 24 h. Finally, the sample was dried in an oven at 220 °C for 24 h.

### 2.4 Synthesis of Cu–BDC@OAC composite

Oxidized activated carbon with terminal –COOH groups was prepared as previously described.^40^ In a typical preparation, commercial coconut shell activated carbon (7 g) was placed in reflux conditions for 4 h in 7.5 M nitric acid. Then, after the reaction is complete, the oxidized carbon is filtered off and washed with de-ionized water to stabilize its pH. Finally, it was dried at 75 °C under vacuum. Obtained samples were marked as oxidized activated carbon (OAC). Similarly, the preparation of the Cu–BDC@OAC composite was carried out by adding oxidized activated carbon (4 g) to well-dissolved MOF precursors (6 mmol from each one) in the same procedure as described for the Cu–BDC followed by continuous stirring for 30 min. The obtained precipitate was washed with DMF for several times and dried in an oven at 220 °C for 24 h.

### 2.5 Characterization

Brunauer–Emmett–Teller (BET) surface area analyzer (Belsorp-Mini II, Gemini 2375 (Bel Japan Inc.)) was used to measure the specific surface area and pore size distributions with N_2_ adsorption/desorption at liquid nitrogen at −196 °C. For achieving this aim, each sample was degassed under the vacuum (10^−5^ torr) at 150 °C for 24 h. The BET surface area (*S*_BET_) and micropore volumes from Dubinin–Radushkevich (*W*_0_) were obtained from the N_2_ adsorption isotherm. Based on the Gurvitch rule, total pore volume was determined, as the N_2_ adsorbed at *P*/*P*_0_ = 0.95. Furthermore, mesopore volume was calculated subtracting the micropore volume to the total pore volume. Also, pore size distributions were determined by applying quenched solid density functional theory (QSDFT) to the N_2_ adsorption data.

The titration method developed by Boehm was applied to study the surface chemistry of AC. The X-ray powder diffraction patterns were recorded on X'pert MPD Philips DW371 with Cu Kα radiation (*λ* = 1.5406 Å). Fourier transform infrared spectroscopy was used to the analysis of the chemical structure and binding features of activated carbon, oxidized activated carbon, MOF and composite (FT-IR, Brucker V33 instrument). The spectra were obtained in the range of 4000–400 cm^−1^, at the resolution of 4 cm^−1^ with 45 scans using the KBr pellet method. The morphologies of samples were also observed by scanning electron microscopy (SEM, TESCAN VEGA-XMU) using ultra-thin coating of gold (Au) with an accelerating voltage of 20 kV with 3000× magnification.

### 2.6 Dynamic adsorption and desorption experiments

The samples were granulated and sieved between 35 and 60 meshes. On a continuous flow type fixed bed reactor, the adsorption and desorption of toluene were performed. The injection technique was used to generate vapors of a particular concentration of toluene.^41^

**Fig. 1:**
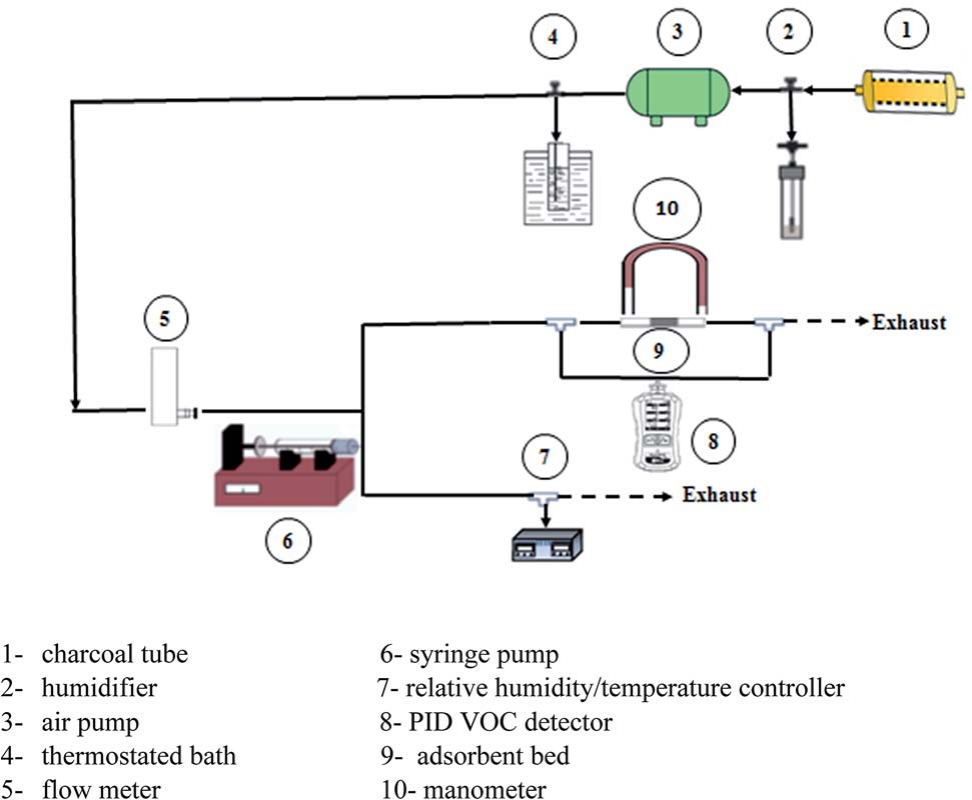
Schematic diagram of the experimental setup.

Fig. 1 shows the schematic diagram of the adsorption and desorption experimental set-up. Firstly, the air produced by a pump passed through a tube containing charcoal to remove potential impurities in the air stream. The flow meter was carried out to control the airflow rate. Toluene vapor was produced by vaporizing toluene flowed out from the syringe pump at a constant rate. The adsorbent was packed in a quartz tube with a 10 mm internal diameter, 12 mm outer diameter and 150 mm long, which MCE (mixed cellulose ester membrane) filter with 37 mm size (SKC) was placed on the top of the reactor as an adsorbent holder. Before any experiment, adsorbents were heated at 110 °C for 2 h in order to remove adsorbed water from the air.

Eqn (1) presents the residence time under this condition:1
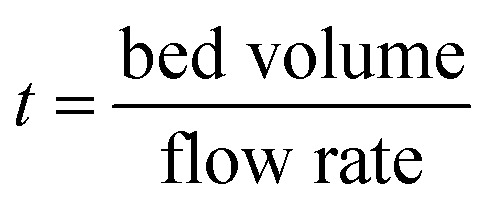


The residence time alters between 0.01 and 0.1 s. The influent and effluent concentrations were measured using a calibrated portable handheld VOC detector equipped with a photoionization detector (MultiRAE, made in the USA). Attention was always paid once the gas mixture was constant at upstream, the adsorption experiment might start.

A humidifier was used to provide the desired relative humidity, which was placed at the upstream. The required temperature was provided using a thermostated bath with a temperature uncertainty of 0.1 °C. The temperature and relative humidity were controlled using ETS electro-tech systems humidity and temperature controller. Breakthrough curves were used to study the activities of the adsorbents toward toluene. The time of the adsorption breakthrough point *t*_b_ and equilibrium *t*_e_ (min) was obtained when the *C*/*C*_0_ reached to 0.1 and 0.5, respectively. The adsorption capacity of adsorbent (mg g^−1^) was as the total mass of the adsorbed toluene per unit weight of adsorbent. It was obtained using eqn (2):^42,43^2
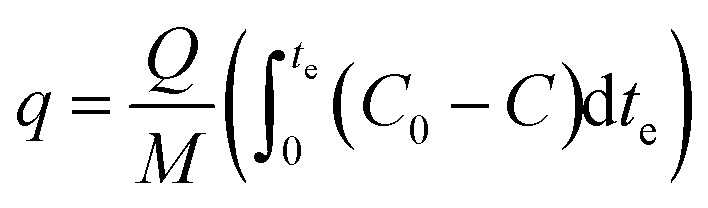
where *C*_0_ and *C* were influent and effluent concentrations of toluene (mg m^−3^). *Q* was the volumetric flow rate (m^3^ h^−1^), *M* was the total mass of adsorbent (g) and *t*_e_ (min) was the time required to reach 50% breakthrough.

For optimization of toluene adsorption, five parameters, including adsorbent mass, flow rate, the concentration of the sample, relative humidity and temperature, were chosen according to preliminary studies and experiments. Because of some drawbacks such as time-consuming and not considering the interaction of the important parameters in the conventional optimization techniques, such as one variable at a time, the response surface methodology (RSM) was selected to design experiments and regulate the process of adsorption. Table 1 presents the selected operational range of parameters.

**Table 1:** Level of independent variables considered in the study of the adsorption of toluene using central composite design (CCD)

Factors	Unit	Symbol	Variable level				
			−2	−1	0	1	2
Adsorbent mass	mg	*x*_1_	15	60	105	150	195
Flow rate	mL min^−1^	*x*_2_	35	90	145	200	255
Adsorbate concentration	ppm	*x*_3_	50	200	350	500	650
Relative humidity	%	*x*_4_	10	30	50	70	90
Temperature	°C	*x*_5_	14	26	38	50	62

Central composite design (CCD) (a full factorial (2^*n*^) of orthogonal type) with 8 central and 10 axial points was used by design expert 11. According to the suggested experimental design, the effective parameters were particularly studied in two sets of 50-run experiments for adsorption of toluene onto the adsorbents. The adsorption capacity of the adsorbents was defined as the response value (*y*).

For assessment of the fitting of results along with the experimental conditions with the response surface models, the main, quadratic and interaction relationships between output responses and independent factors were studied using a second-order polynomial model according to the eqn (3):3
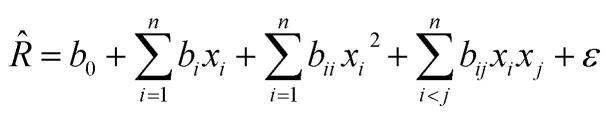
where *R̂* is the predicted response, *x*_*i*_ is variables, *b*_0_, *b*_*i*_, *b*_*ii*_, *b*_*ij*_ are the regression coefficients and *ε* is the statistical error.

The adequacy of the response surface quadratic model was justified through the analysis of variance (ANOVA). The model *F*-value was considered to assess the statistical significance of the model. Statistical *F*-value with a low ‘*P*’ value demonstrates a high significance of the regression model.

*R* ^2^ and adjusted *R*^2^ (the ANOVA coefficient of determination) was used to express the quality of fit for the polynomial model equation. According to the results, the two values were both close to 1, which showed a moderately high level of correlation between the actual and the predicted responses. Finally, the model can be acceptable for data when the *P*-value for lack of fit in the ANOVA tests is higher than 0.05.

## 3. Results and discussion

### 3.1 Characterization of Cu–BDC@OAC composite

**Fig. 2:**
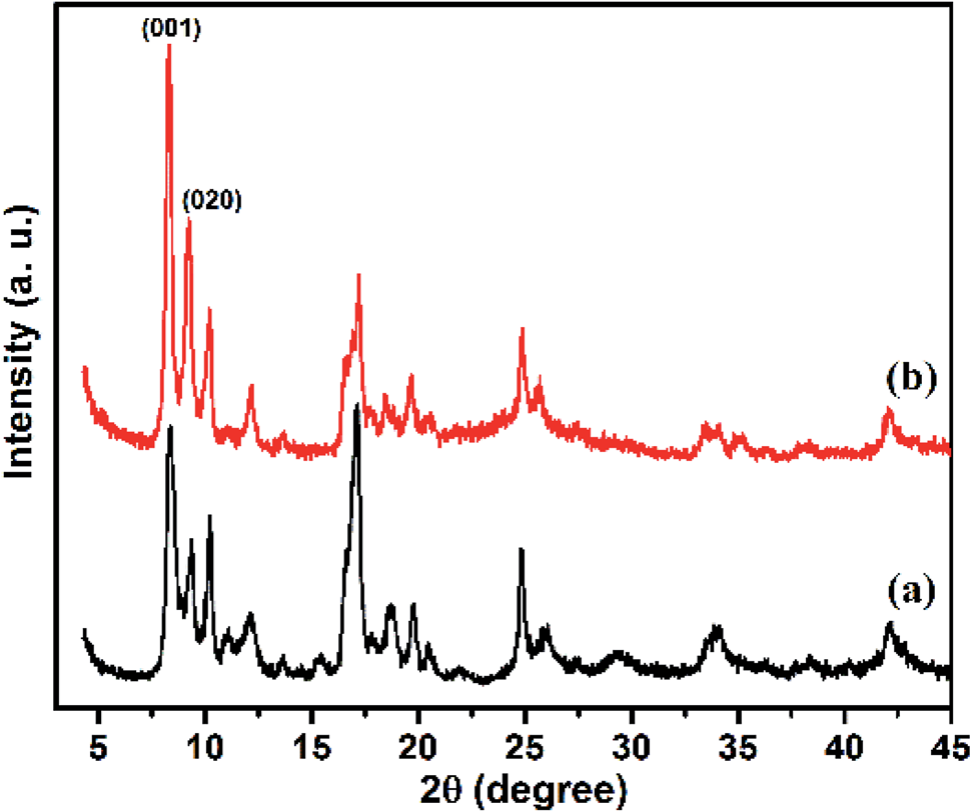
The XRD patterns of (a) Cu–BDC MOF and (b) Cu–BDC@OAC composite.

As shown in Fig. 2, the XRD patterns of Cu–BDC MOF and Cu–BDC@OAC composite are presented, comparatively. Fig. 2a represents the XRD pattern of the Cu–BDC with sharp peaks, which are consistent with the diffraction peaks centered at 2*θ* = 8.3° and 9.1° representing (001) and (020) planes of crystal structure respectively, match well with relevant reported in the literature.^19^ The high intensity of the peaks in the XRD pattern demonstrated that the good crystallization of Cu–BDC MOF was developed. Also, the characteristic peaks of Cu–BDC MOF are observed in the composite based on oxidized activated carbon, which confirms the successful formation of Cu–BDC@OAC composite. The appearance of the two main peaks at (2*θ* = 8.3° and 9.1°) less than 2*θ* = 10° in both Cu–BDC MOF and Cu–BDC@OAC indicates that the DMF solvent completely evaporated at 220 °C.^44^

**Fig. 3:**
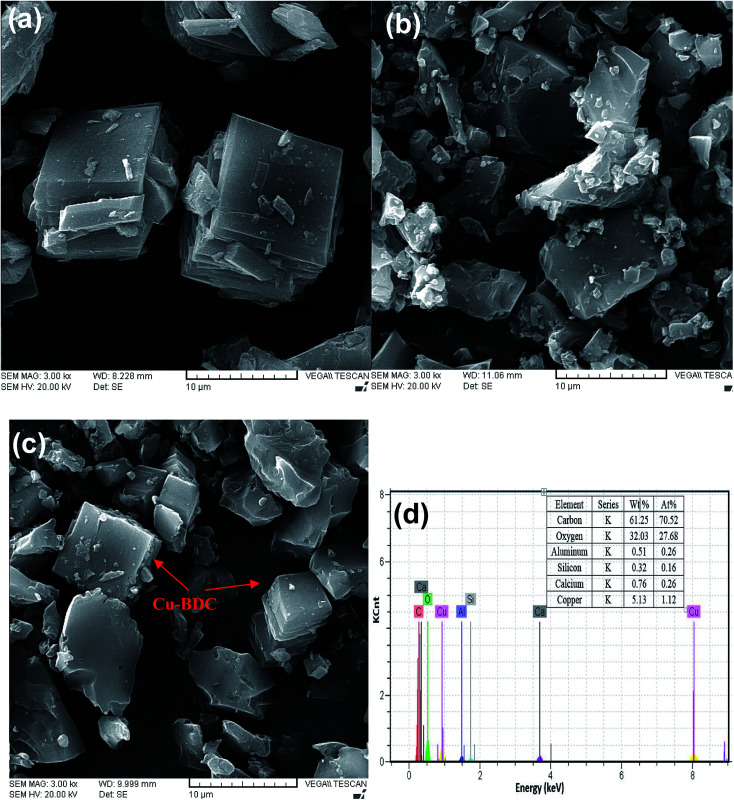
SEM images of (a) Cu–BDC, (b) oxidized activated carbon (c) Cu–BDC@OAC composite and (d) EDS spectra of Cu–BDC@OAC composite.

The SEM images of the as-synthesized Cu–BDC, oxidized activated carbon and Cu–BDC@OAC composite are shown in Fig. 3 respectively. The SEM image shows that the Cu–BDC MOF particles are uniform with well-formed cubic microcrystals and, which are in agreement with their XRD patterns and their average edge length is about 9.3 μm. Its morphology was compatible with the previous results that were achieved from hydrothermal synthesis.^39,45^Fig. 3b shows that the oxidized activated carbon is amorphous and its particle size is below 10 μm. Comparing the SEM images of oxidized activated carbon and Cu–BDC@OAC composite (Fig. 3b and c) the successful formation of crystalline Cu–BDC on oxidized activated carbon can be observed. Furthermore, the EDS spectra and elemental analysis data (Fig. 3d) show that the Cu–BDC@OAC composite has been successfully synthesized and it is confirmed by the presence of Cu.

**Fig. 4:**
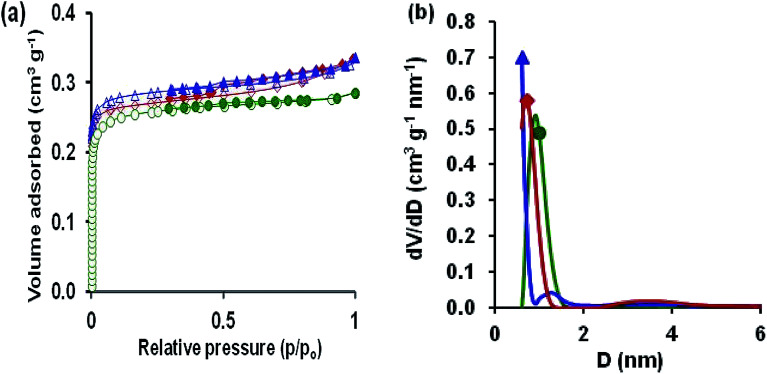
(a) N_2_ adsorption–desorption isotherms and (b) QSDFT pore size distribution of oxidized activated carbon, 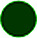; Cu–BDC, 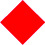 and Cu–BDC@OAC composite, 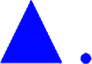. Open symbols adsorption branch, closed symbols desorption branch.

The specific textural properties of oxidized AC, Cu–BDC and Cu–BDC@OAC composite were determined by N_2_ adsorption–desorption measurements at −196 °C (Fig. 4a). According to the IUPAC nomenclature, the illustrated isotherm of oxidized AC was type I, which is typical for microporous materials.

The specific BET surface area obtained for the oxidized AC is 632 m^2^ g^−1^, and the total volume of pores is 0.277 cm^3^ g^−1^. Cu–BDC and Cu–BDC@OAC composite revealed the isotherm type I with H2 hysteresis, which is related to microporous materials with a BET surface area of 686 and 712 m^2^ g^−1^ respectively. In the N_2_ adsorption–desorption isotherms of Cu–BDC and Cu–BDC@OAC composite, there is a small hysteresis loop, which can be attributed to the H2-type hysteresis loop, indicating the presence of mesopores.^46^ Larger micropore volume of Cu–BDC@OAC sample leads to a higher N_2_ adsorption capacity at low pressures. As shown in Fig. 4a, Cu–BDC and Cu–BDC@OAC have higher N_2_ adsorption capacity at high pressures because of their larger *V*_total_ (Table 2). Also, there has been some increase in the specific surface area of Cu–BDC@OAC composite even more than pure Cu–BDC, which could be due to the synergistic action.^30,37,47^ Pore size distribution (PSD) demonstrates a model of solid internal structure, which shows that a similar set of non-interacting and commonly shaped model pores can indicate the complex void spaces. The PSD is nearly attributed to both kinetic and equilibrium features of porous material, and it can be the most significant factor to characterize the structural heterogeneity of porous materials. Fig. 4b shows the pore size distributions of the adsorbents derived from QSDFT. Therefore, the prepared Cu–BDC@OAC composite has mainly micropores with some mesopores. The presence of the uplift curve at diameter < 2.5 nm for Cu–BDC@OAC proves the existence of micropore structure.^48^ Also, the BET surface areas and different textural parameters of the samples are listed in Table 2. From this table, it can be found that introducing Cu–BDC MOF to OAC caused a significant effect on the textural features (Cu–BDC@OAC).

**Table 2:** Textural parameters of the samples

Sample	*S*_BET_	*S*_DFT_	*V*_micro_	*V*_meso_	*V*_total_
	(m^2^ g^−1^)	(m^2^ g^−1^)	(cm^3^ g^−1^)	(cm^3^ g^−1^)	(cm^3^ g^−1^)
Oxidized AC	632	523	0.260	0.017	0.277
Cu–BDC	686	707	0.269	0.066	0.335
Cu–BDC@OAC	712	966	0.287	0.049	0.336

**Fig. 5:**
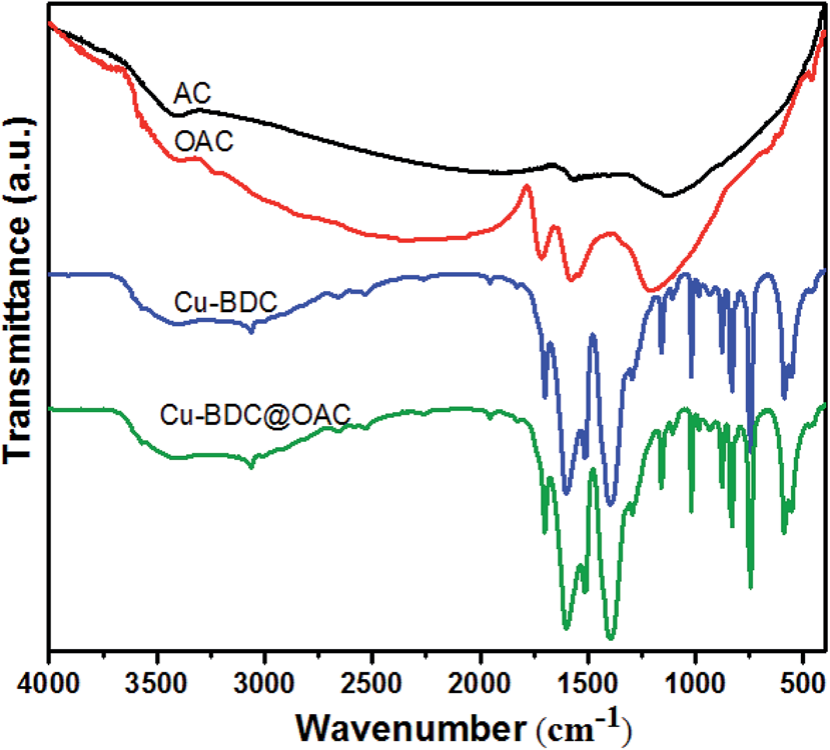
FT-IR spectra of activated carbon, oxidized AC, Cu–BDC and Cu–BDC@OAC composite.

The FT-IR spectra of the activated carbon, oxidized AC, Cu–BDC and Cu–BDC@OAC composite were displayed in Fig. 5. There is a clear difference in the IR spectrum between the oxidized AC compared to the original activated carbon after the oxidation with nitric acid. Typical bands of the oxidized AC at 1716 cm^−1^ can be assigned to the stretching vibration from ketones, or carboxyl groups. Also, the 1577 cm^−1^ band belongs to the aromatic ring conjugated with C=O carbonyl groups.^40^ The broad peak around the 3400 cm^−1^ represents the carboxylic OH group. The 1185 cm^−1^ peak corresponded to C–O stretching vibration and OH bending mode of alcoholic, phenolic and carboxylic groups. In the as-synthesized Cu–BDC spectra, the sharp absorption peaks at 1602 and 1398 cm^−1^ belong to the asymmetric and symmetric stretching modes of the carboxylate groups, respectively. The bands at 1512 and 743 cm^−1^ are assigned to the vibrations of the phenyl ring. The vibration bands at 468 and 556 cm^−1^ can be considered as the stretching vibration peaks of Cu–O.^39^ Also, there are no solvent (DMF) absorption peaks, which is in agreement with the XRD patterns of these samples. All of the mentioned peaks of Cu–BDC are observed in the infrared spectrum of the Cu–BDC@OAC composite without shift.

Notably, the peak 1716 cm^−1^ for the oxidized AC shifts to a low wavenumber or disappears, which may be due to bond formation between Cu ions in Cu–BDC MOF and –COO groups on the oxidized AC surface.

The Boehm titration revealed that the quantities of carboxylic, lactonic and phenolic groups are, respectively, 1.7, 0.55 and 1.25 meq g^−1^ for oxidized AC. The surface of the original activated carbon does not contain a carboxylic group and is approximately zero. Carboxylic groups are the higher acidity, followed by phenolic groups, and then lactonic groups.

**Fig. 6:**
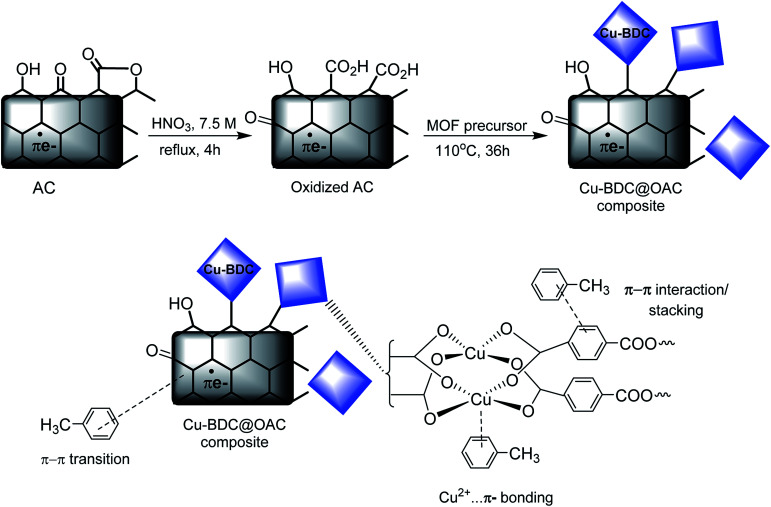
Schematic of synthesizing of Cu–BDC MOF on the oxidized AC surface and the proposed mechanism of toluene adsorption on the Cu–BDC@OAC composite adsorbent.

Fig. 6 depicts the schematic synthesis of Cu–BDC on the oxidized AC surface and the proposed mechanism of toluene adsorption on the Cu–BDC@OAC composite adsorbent. After the oxidation reaction on the surface of activated carbon with HNO_3_, the proportion of acidic functional groups, particularly the carboxylic acid (–COOH) groups, was escalated on the surface of AC (approved by Boehm titration). After adding oxidized activated carbon to well-dissolved MOF precursors, the chemical bond is formed between Cu^2+^ ions in MOF and –COO groups on the surface of oxidized activated carbon due to the existence of vacant coordination sites in Cu(ii).^49^ Also, the proposed mechanism of toluene adsorption is presented in Fig. 6. Pore-filling is proposed to be an important mechanism participating in the micropore of activated carbon and Cu–BDC in the synthesized composite. Furthermore, Cu–BDC@OAC has other types of adsorption mechanisms, which include transition metal cation–π interactions, π–π stacking, donor–acceptor complexes with the C=O group (–COOH) and hydrophobic interactions from phenyl ring in the wall of the structure of MOF. Cu^2+^ cation could form π-complex bonds with toluene molecules, creating more active adsorption sites, which greatly enhanced the selectivity of adsorption (confirmed by the adsorption tests).^50^

### 3.2 Toluene adsorption studies

#### 3.2.1 Dynamic adsorption behaviors

In order to study the adsorption behavior of the composite toward toluene, adsorption capacity, and breakthrough points resulting from column tests were employed in a continuous mode. The best adsorption capacity and breakthrough point of Cu–BDC@OAC composite were 220.7 mg g^−1^ and 152.8 min, respectively. The comparison of the adsorption capacity for toluene vapor by adsorbents reported in the literature is presented in Table 3. The present microporous Cu–BDC@OAC composite demonstrates the better performance of toluene adsorption than many kinds of adsorbents, which recommends that the microporous Cu–BDC@OAC composite is an efficient toluene adsorbent. The microporous Cu–BDC@OAC composite acquires good potential for toluene removal in air purification. The current results showed that the volume of micropores was an important factor for the removal of VOCs and are consistent with those reported in the literature.^48,51,52^ Based on the theory of volume filling of micropores, adsorption is increased because of the overlap of dispersion forces from adjacent pore walls.^51^

**Table 3:** Toluene adsorption capacities of different adsorbents

Adsorbents	Experimental conditions^a^	Toluene adsorption capacity (mg g^−1^)	Reference
Cu–BDC@OAC	*T*: 26, RH: 30, *Q*: 90, *C*_0_: 500	220.7	This study
Cu-BTC@ZSM-5^b^	*T*: 25, RH: 30, *Q*: 180, *C*_0_: ∼345	158.6	52
ASMS^c^	*T*: 35, *Q*: 60, *C*_0_: 2	98.1	53
CPM-5^d^	*T*: 25, RH: 30, *Q*: 600, *C*_0_: 1	50	8
SWCNT (NaOCl)^e^	*T*: 25, *C*_0_: 200	103.2	54
ACF^f^	*T*: 20, *C*_0_: ∼100	85	55

a *T* = temperature (°C); RH = relative humidity (%); *Q*; flow rate (mL min^−1^); *C*_0_ = initial toluene concentration (ppm).

b Copper–benzene-1,3,5-tricarboxylic acid@Zeolite Socony Mobil-5.

c Amino-functionalized spherical mesoporous silica.

d Crystalline porous material.

e Single-walled carbon nanotube.

f Activated carbon fiber.

**Fig. 7:**
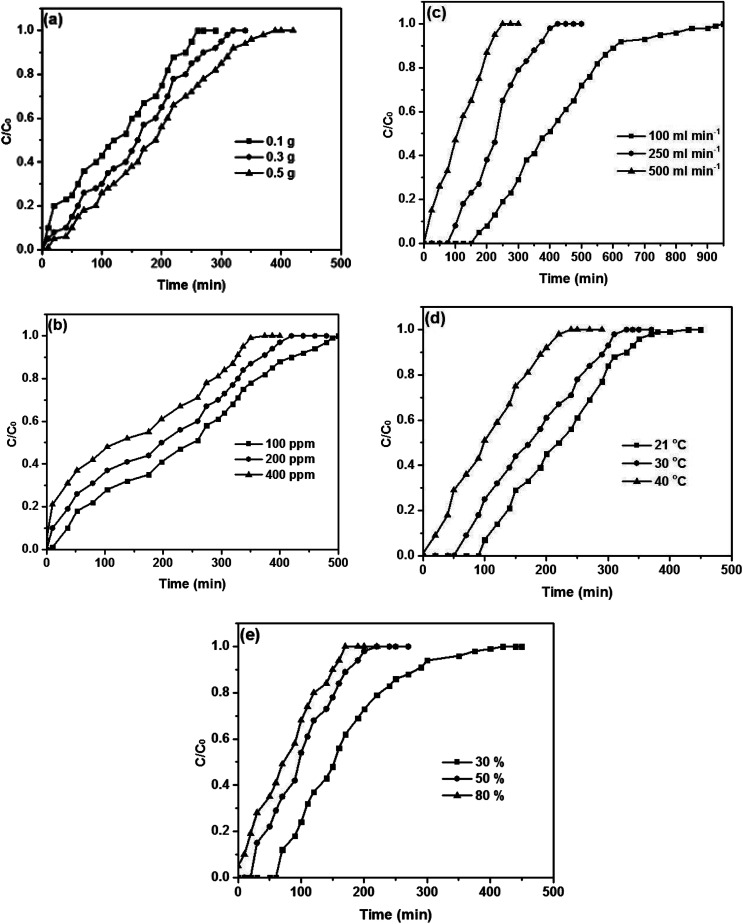
Breakthrough curves of toluene on the Cu–BDC@OAC composite at different (a) adsorbent masses, (b) concentrations, (c) flow rates, (d) temperatures and (e) relative humidity levels.

The effects of different operational parameters for adsorption of toluene on the composite were studied separately. The effect of adsorbent mass on the adsorption capacity in order to determine the required adsorbent quantity for maximum removal of toluene was investigated and illustrated in Fig. 7a. Adsorbent mass studies were done at temperature 40 °C, flow rate 150 mL min^−1^ and initial concentration 350 ppm with relative humidity 50% for three different adsorbent masses (100–300–500 mg). At first glance, it is evident that the breakthrough point of the adsorbent declines with the increasing weight of the adsorbent. The adsorption capacity declined from 195 mg g^−1^ to 149.5 mg g^−1^ when the weight of the adsorbent was increased from 100 mg to 500 mg. This can be interpreted as the higher the mass of adsorbent, the greater the accessibility of the interchangeable sites or surface offered to the adsorption of toluene.^28,47^

The influence of the initial concentration on the adsorption capacity of the adsorbent was examined. The experiments were performed at various initial concentrations (100–200–400 ppm) at the fixed adsorbent mass of 100 mg, temperature 40 °C, flow rate 150 mL min^−1^ and relative humidity 50%. The adsorption capacity decreased with an increase in initial concentration (Fig. 7b). The adsorption capacity reduced from 198.9 mg g^−1^ to 178.6 mg g^−1^ when the toluene concentration was increased from 100 ppm to 400 ppm. The toluene concentration influences the diffusion of toluene molecules to the surface of adsorbent since agitation speed is constant. An increase of the toluene concentration accelerates the diffusion of toluene molecules onto the adsorbent as a result of the increase in the driving force of the concentration gradient.^56,57^

The effect of flow rate on the adsorption of toluene is depicted in Fig. 7c. The flow rate experiments were conducted at three different flow rates of air passing from adsorbent at a temperature of 40 °C, the relative humidity of 50% and initial concentration of 350 ppm for a constant adsorbent mass of 100 mg. The adsorption capacity of toluene was found to decline with increasing flow rate. Also, when the flow rate increased, the breakthrough curve becomes steeper. With increasing flow rate, the adsorption capacity is decreased due to the increase in the velocity of passing airstream over the composite surface; so, whether the residence time of the toluene in the reactor is not long sufficient for adsorption equilibrium to be reached at that flow rate, the toluene vapor leaves the reactor before equilibrium happens. Thence, at a higher flow rate, the contact time of toluene ions with the adsorbent is very short so that the adsorption capacity will be declined.^28,58^

The relationship between temperature and adsorption capacity was illustrated in Fig. 7d. The experiments were done for an initial concentration of 350 ppm at a flow rate 150 mL min^−1^ and relative humidity 50% with constant carbon loading of adsorbent mass of 100 mg. The results reveal that the amount of toluene adsorbed declined moderately with increasing temperature from 21 °C to 40 °C. The decrease in the adsorption capacity of toluene indicates that the significance of the physisorption mechanism between the composite and gaseous toluene.^47,59^

Fig. 7e illustrates the relationship between humidity and adsorption capacity. Humidity experiments were executed at temperature 40 °C, flow rate 150 mL min^−1^ and initial concentration 350 ppm at the fixed adsorbent mass of 100 mg for three different relative humidity ranges (30–50–80%). It is evident from Fig. 7e that the presence of relative humidity (RH) has a significant negative impact on the rate of toluene adsorption on the adsorbent, demonstrating a competitive adsorption process between toluene and water vapor. The amount of adsorption capacity declines relatively in the presence of 30% RH, and this proportion drops noticeably in the presence of 80% RH. The structure of the composite could be deformed due to interactions between hydrogens in H_2_O and oxygens in carboxylate, so it hindered toluene from reaching the active sites of the adsorbent.^47,60,61^

#### 3.2.2 The optimization of adsorption conditions

Herein, the RSM was applied to optimize the adsorption of toluene in the airstream using adsorbents. In this section, the microporous Cu–BDC@OAC composite was investigated. Table 4 presents the experimental design used for the investigation of toluene adsorption over the composite. The values in adsorption presented in Table 5 indicate the validity of the models to predict the response. The multivariate regression model was used to predict the toluene adsorption through the dynamic method using the quadratic models, which was presented to the following form in the eqn (4):

4 Adsorption capacity (*Ŷ*) = 285.48816 − 0.452216*X*_1_ − 0.235563*X*_2_ − 0.029443*X*_3_ − 0.105717*X*_4_ − 1.37796*X*_5_ + 0.000156*X*_1_*X*_2_ − 0.000015*X*_1_*X*_3_ − 0.000042*X*_1_*X*_4_ + 0.001591*X*_1_*X*_5_ − 0.000034*X*_2_*X*_3_ − 0.000022*X*_2_*X*_4_ − 0.000208*X*_2_*X*_5_ − 0.000452*X*_3_*X*_4_ + 0.000291*X*_3_*X*_5_ − 0.001471*X*_4_*X*_5_ + 0.001461*X*_1_^2^ 0.000700*X*_2_^2^ + 0.000092*X*_3_^2^ − 0.000618*X*_4_^2^ + 0.012702*X*_5_^2^

**Table 4:** Adsorption capacity and breakthrough time of the microporous Cu–BDC@OAC composite in different operating conditions

Run no.	Std	Type	Adsorbent mass (mg)	Flow rate (mL min^−1)^	Concentration (ppm)	Temperature (°C)	Relative humidity (%)	Adsorption capacity (mg g^−1^)	Breakthrough time (min)
1	17	Factorial	60	90	200	50	30	205.38	36.33
2	16	Factorial	150	200	500	26	70	192.11	15.29
3	14	Factorial	150	90	500	26	70	196.44	34.75
4	35	Center	105	145	350	38	50	190.44	20.91
5	27	Factorial	60	200	200	50	70	187.55	14.93
6	21	Factorial	60	90	500	50	30	215.04	15.21
7	38	Center	105	145	350	38	50	190.44	20.91
8	36	Center	105	145	350	38	50	190.44	20.91
9	37	Center	105	145	350	38	50	190.44	20.91
10	12	Factorial	150	200	200	26	70	189.92	37.79
11	11	Factorial	60	200	200	26	70	197.55	15.72
12	19	Factorial	60	200	200	50	30	199.7	15.9
13	24	Factorial	150	200	500	50	30	206.66	16.45
14	15	Factorial	60	200	500	26	70	199.47	6.35
15	28	Factorial	150	200	200	50	70	184.74	36.76
16	26	Factorial	150	90	200	50	70	186.76	82.59
17	5	Factorial	60	90	500	26	30	220.79	15.62
18	34	Center	105	145	350	38	50	200.44	22
19	3	Factorial	60	200	200	26	30	208.5	16.6
20	10	Factorial	150	90	200	26	70	194.34	85.95
21	22	Factorial	150	90	500	50	30	210.56	37.24
22	4	Factorial	150	200	200	26	30	202.4	40.28
23	33	Center	105	145	350	38	50	190.44	20.91
24	18	Factorial	150	90	200	50	30	200.39	88.62
25	2	Factorial	150	90	200	26	30	204.11	90.27
26	7	Factorial	60	200	500	26	30	215.4	6.86
27	29	Factorial	60	90	500	50	70	197.33	13.42
28	8	Factorial	150	200	500	26	30	209.49	16.67
29	1	Factorial	60	90	200	26	30	211.63	37.44
30	6	Factorial	150	90	500	26	30	213.46	37.76
31	13	Factorial	60	90	500	26	70	203.24	14.38
32	9	Factorial	60	90	200	26	70	201.06	35.56
33	20	Factorial	150	200	200	50	30	197.76	39.35
34	30	Factorial	150	90	500	50	70	192.95	34.13
35	32	Factorial	150	200	500	50	70	189.67	15.09
36	31	Factorial	60	200	500	50	70	190.62	6.07
37	25	Factorial	60	90	200	50	70	192.33	34.02
38	23	Factorial	60	200	500	50	30	209.41	6.66
39	46	Axial	105	145	350	38	90	175.61	19.28
40	40	Axial	195	145	350	38	50	201.87	41.16
41	41	Axial	105	35	350	38	50	208.52	94.86
42	44	Axial	105	145	650	38	50	209.23	12.37
43	45	Axial	105	145	350	38	10	213.95	23.49
44	49	Center	105	145	350	38	50	195.79	21.49
45	48	Axial	105	145	350	62	50	196.64	21.59
46	50	Center	105	145	350	38	50	195.79	21.49
47	47	Axial	105	145	350	14	50	209.53	23
48	39	Axial	15	145	350	38	50	213.34	3.34
49	43	Axial	105	145	50	38	50	174.83	152.83
50	42	Axial	105	255	350	38	50	199.96	12.48

**Table 5:** ANOVA data for the toluene adsorption over the microporous Cu–BDC@OAC composite^a^

Source	Sum of squares	Degrees of freedom	Mean square	*F* value	*P* value
Model	4332.60	20	216.63	48.01	<0.0001
*X*_1_	281.85	1	281.85	62.46	<0.0001
*X*_2_	168.02	1	168.02	37.23	<0.0001
*X*_3_	355.93	1	355.93	78.88	<0.0001
*X*_4_	2422.38	1	2422.38	536.83	<0.0001
*X*_5_	353.07	1	353.07	78.25	<0.0001
*X*_1_ *X*_2_	4.76	1	4.76	1.05	0.3132
*X*_1_ *X*_3_	0.3444	1	0.3444	0.0763	0.7844
*X*_1_ *X*_4_	0.0450	1	0.0450	0.0100	0.9212
*X*_1_ *X*_5_	4	1	23.63	5.24	0.0299
*X*_2_ *X*_3_	2.59	1	2.59	0.5735	0.4552
*X*_2_ *X*_4_	0.0190	1	0.0190	0.0042	0.9487
*X*_2_ *X*_5_	0.6050	1	0.6050	0.1341	0.7170
*X*_3_ *X*_4_	58.75	1	58.75	13.02	0.0012
*X*_3_ *X*_5_	8.76	1	8.76	1.94	0.1746
*X*_4_ *X*_5_	3.99	1	3.99	0.8843	0.3551
*X*_1_^2^	277.64	1	277.64	61.53	<0.0001
*X*_2_^2^	142.22	1	142.22	31.52	<0.0001
*X*_3_^2^	135.25	1	135.25	29.97	<0.0001
*X*_4_^2^	1.94	1	1.94	0.4294	0.5176
*X*_5_^2^	106.08	1	106.08	23.51	<0.0001
Residuals	126.35	28	4.51	—	—
Lack of fit	43.01	22	1.96	0.1408	0.9997
Pure error	83.33	6	13.89	—	—

a *R* ^2^ = 0.9717; adjusted *R*^2^ = 0.9514; predicted *R*^2^ = 0.9260.

*R* ^2^ = 0.9717, which is for eqn (4), demonstrates that almost 2.83% of the whole alterations were not adequately explained by the model. From Table 5, it is immediately apparent that the statistical *F* value of the model was high. This great value represents that the adsorption capacity can be acceptably explained by the model. The residual error is the unexplained part of the model (4.51), which is presented in Table 5. It was seen that the linear effects of all the factors were significant (*P* < 0.001) and the coefficient of *X*_1_^2^, *X*_2_^2^, *X*_3_^2^ and *X*_5_^2^ were considerably significant (*P* < 0.001) in comparison with the interaction terms *X*_1_*X*_5_ (*P* = 0.0299) and *X*_3_*X*_4_ (*P* = 0.0012). The most important term on the adsorption capacity was *X*_4_ with *F*-value 536.83.

**Fig. 8:**
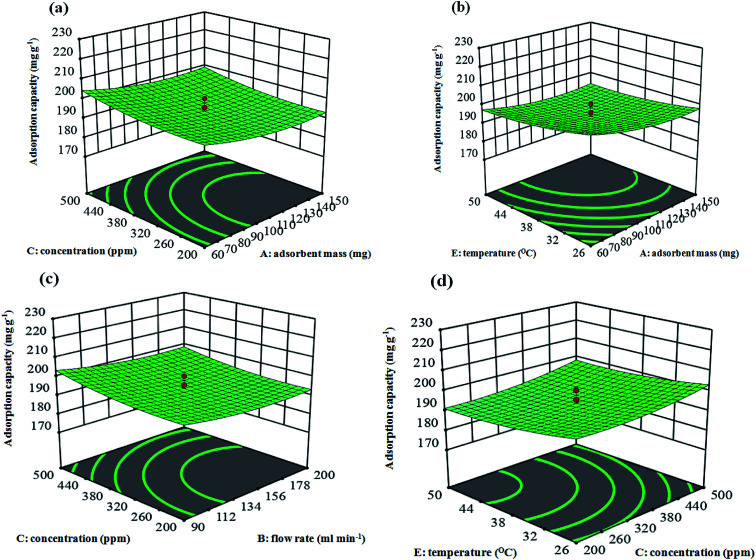
(a) 3D response surface: interactive effects of adsorbent mass and concentration at a flow rate of 145 mL min^−1^, 40 °C and relative humidity 50% on adsorption capacity. (b) 3D response surface: interactive effects of adsorbent mass and temperature at concentration 350 ppm, flow rate 145 mL min^−1^ and relative humidity 50% on adsorption capacity. (c) 3D response surface: interactive effects of flow rate and concentration at adsorbent mass 105 mg, temperature 40 °C and relative humidity 50% on adsorption capacity. (d) 3D response surface: interactive effects of concentration and temperature at adsorbent mass 105 mg, flow rate 145 mL min^−1^ and relative humidity 50% on adsorption capacity.

The behavior of the response surface models can be shown using graphical representations of the parameter dependencies, such as 3D response plots, where the interaction influence of the parameters have been mapped against the response factors. The adsorption capacities of the adsorbent (the Cu–BDC@OAC) over the various mixtures of independent parameters were therefore shown through plots (Fig. 8a–d). In this way, the plots were illustrated as a function of two parameters at a time, maintaining other variables at a fixed state. The response surfaces at low and high levels of the parameters lead to minimal capacity of the adsorbent. The results show that there is an area where neither an increasing nor a decreasing trend in the adsorption capacity is observed. This trend demonstrates an optimum point for the adsorption parameters to enhance the adsorption capacity. Additionally, it is worth noting that there was a moderate interaction between the adsorbent mass and temperature (*X*_1_ and *X*_5_) (*P* = 0.0299) and concentration and relative humidity (*X*_3_ and *X*_4_) (*P* = 0.0012) on the toluene adsorption (Fig. 8b).

Several optimum points were suggested, including the factors along with the predicted response value using the CCD *via* model confirmation (Table 6). The experimental findings were in good agreement with the predicted results because there was a negligible error between predicted and experimental results, and “desirability” values were attained up to 100%, indicating the great closeness of response (*y*) to their ideal values. So, the model for the optimization of toluene adsorption over the adsorbent in this survey obtained high compatibility and hence could be applied to design and optimize the experimental conditions.^62^

**Table 6:** The optimum point for adsorption process through point prediction method

Solution no.	Adsorbent mass (mg)	Concentration (ppm)	Flow rate (mL min^−1^)	Temperature (°C)	Relative humidity (%)	Adsorption capacity (mg g^−1^)		Desirability
						Predicted	Experimental	
1	**60.000**	**500.000**	**90.000**	**26.000**	**30.000**	**222.811**	**220.79**	**1.000**
2	61.885	494.524	90.064	26.450	30.391	221.709	219.60	1.000
3	62.336	486.535	90.950	26.025	30.170	221.405	219.33	1.000
4	60.099	499.773	90.150	26.123	33.753	221.140	219.17	1.000
5	60.011	465.943	90.051	26.008	30.024	221.050	218.82	1.000

**Fig. 9:**
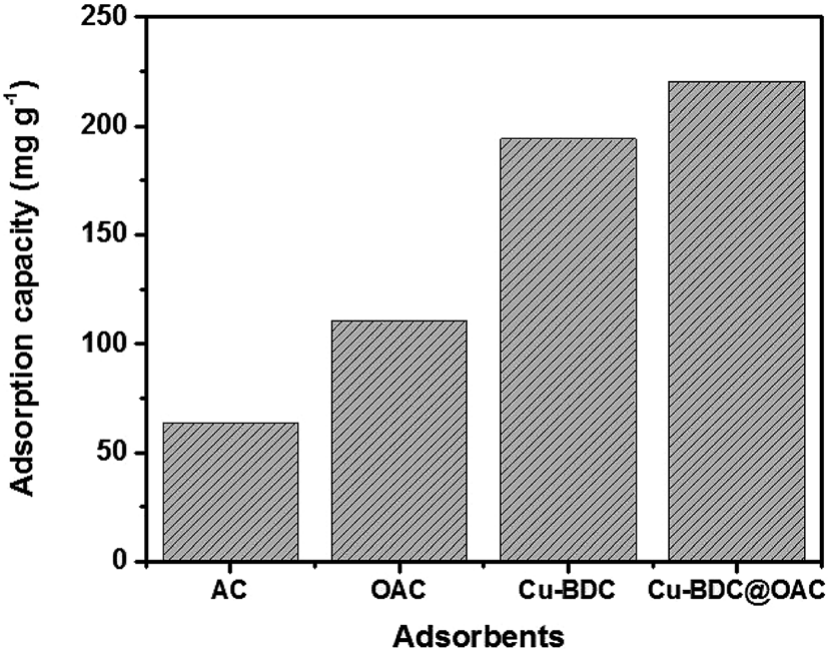
Comparison of the highest adsorption capacity values in the optimized process conditions by adsorbents.

The obtained highest adsorption capacities (mg g^−1^) of gaseous toluene over activated carbon (AC), oxidized activated carbon (OAC), Cu–BDC and the Cu–BDC@OAC composite in the optimized process conditions were depicted in Fig. 9. It is clearly shown that the Cu–BDC@OAC composite has the highest adsorption capacity in comparison with the rest. In previous surveys, Bingman Lei *et al.* did a comparative study about the adsorption capacity of CuO-modified activated carbon for the improvement of toluene removal from air. The adsorption capacity of CuO/AC composite was better than for AC (1.2–1.9-fold higher than those of AC). The equilibrium adsorption capacity of CuO/AC 0.3 wt% composite (AC03) was 701.8 mg g^−1^.^27^ Zhang *et al.*, reported the adsorption capacities of UiO-66, CTAB-U-0.3, CTAB-U-0.5 and CTAB-U-1 were 151, 177, 275 and 204 mg g^−1^,^47^ respectively.

#### 3.2.3 The regenerability of the microporous Cu–BDC@OAC composite

**Fig. 10:**
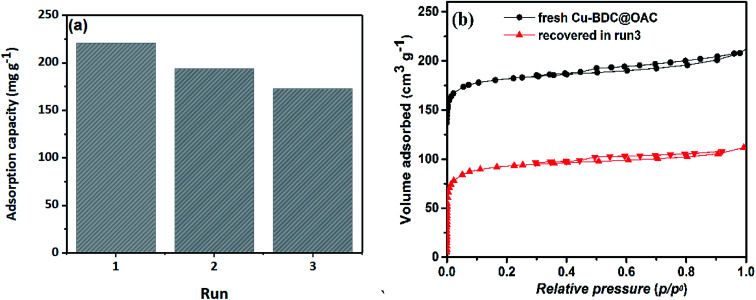
(a) Regeneration performance of Cu–BDC@OAC (b) N_2_ adsorption–desorption isotherms of the fresh and recovered Cu–BDC@OAC.

The reusability performance of adsorbent was a significant factor in investigating the application of adsorbents. Toluene was desorbed using a temperature-programmed desorption process following the dynamic toluene adsorption and, therefore, re-adsorbed for three thermal regeneration cycles at 150 °C in N_2_ flow for 4 h to evaluate the reversibility of toluene adsorption on the microporous Cu–BDC@OAC composite. Fig. 10a illustrates the regenerability of toluene adsorption on the adsorbent. The adsorption capacity of toluene over the adsorbent at the third run was 172.9 mg g^−1^, which is almost 78% of the initial values. The microporous Cu–BDC@OAC composite showed good recyclability and stability and had the potential to be a suitable adsorbent for toluene removal. Furthermore, Fig. 10b shows the N_2_ adsorption–desorption isotherms of the fresh and recovered adsorbent (run 3), which confirms the stability of the porous structure. The BET surface area decreased from 712 m^2^ g^−1^ in the fresh sample to 352 m^2^ g^−1^, which may be due to a blockage or destruction of some pores.

## 4. Conclusions

In summary, the microporous Cu–BDC@OAC composite was synthesized by *in situ* incorporation of the Cu–BDC MOF to oxidized activated carbon with terminal –COOH groups. Characterization of this adsorbent showed the larger specific surface area (712 m^2^ g^−1^) than that of Cu–BDC MOF and oxidized AC, respectively. Dispersed Cu–O bonds combined with hydrophobic phenyl groups and oxygen-containing functional groups of oxidized AC lead to enhance the surface affinity to toluene and prepare more active adsorption sites. The pore structure of adsorbents was identified to play an important role in the adsorption features of toluene. Micropores appeared a positive effect on the adsorption of toluene. Optimization based on the statistical design of experiments was shown to be a useful tool for predicting and investigating the interaction effects of experimental factors. RSM and the CCD were proper for determining the optimal conditions for toluene adsorption onto the Cu–BDC@OAC composite. An adsorbent mass of 60 mg, a flow rate of 90 mL min^−1^, a concentration of 500 ppm, relative humidity of 30% and a temperature of 26 °C were found to be the optimized process conditions. The maximum adsorption capacity of toluene onto Cu–BDC@OAC composite was 222.811 mg g^−1^, which increased by almost 12% and 50% compared with pure Cu–BDC and oxidized AC, respectively. The regeneration of the composite was still up to 78% after three consecutive adsorption–desorption cycles. The higher adsorption capacity and reusability make the microporous Cu–BDC@OAC composite a superior VOCs adsorbent.

## Conflicts of interest

The authors state that there is no conflict of interest in this study.

## Supplementary Material
